# Pulmonary Paragonimiasis: A Pitfall in IgG4-Related Disease Diagnosis

**DOI:** 10.4269/ajtmh.25-0112

**Published:** 2025-05-27

**Authors:** Hidenori Takahashi, Takumi Yasuda, Mio Kokubo-Tanaka

**Affiliations:** ^1^ Department of Respiratory Medicine, Tokyo Shinagawa Hospital, Tokyo, Japan;; ^2^ Division of Parasitology, Department of Infectious Diseases, Faculty of Medicine, University of Miyazaki, Miyazaki, Japan

A 50-year-old man, originally from Hong Kong and residing in Japan, was referred for further evaluation of abnormal lung shadows detected during a routine health checkup after a persistent productive cough owing to suspected lung cancer. The patient regularly consumed raw meat, including wild game, but never raw crabs. Chest computed tomography (CT) showed irregular nodular opacities (24 mm) in both upper lobes with increased fluorodeoxyglucose uptake on positron emission tomography but no lymphadenopathy or pleural effusion (Figure 1A and B). Laboratory tests revealed marked eosinophilia (eosinophils, 1,580/*µ*L; 20.2%) and elevated IgG4 levels (491 mg/dL), with normal total IgG levels (1,375 IU/mL).

**Figure 1.**
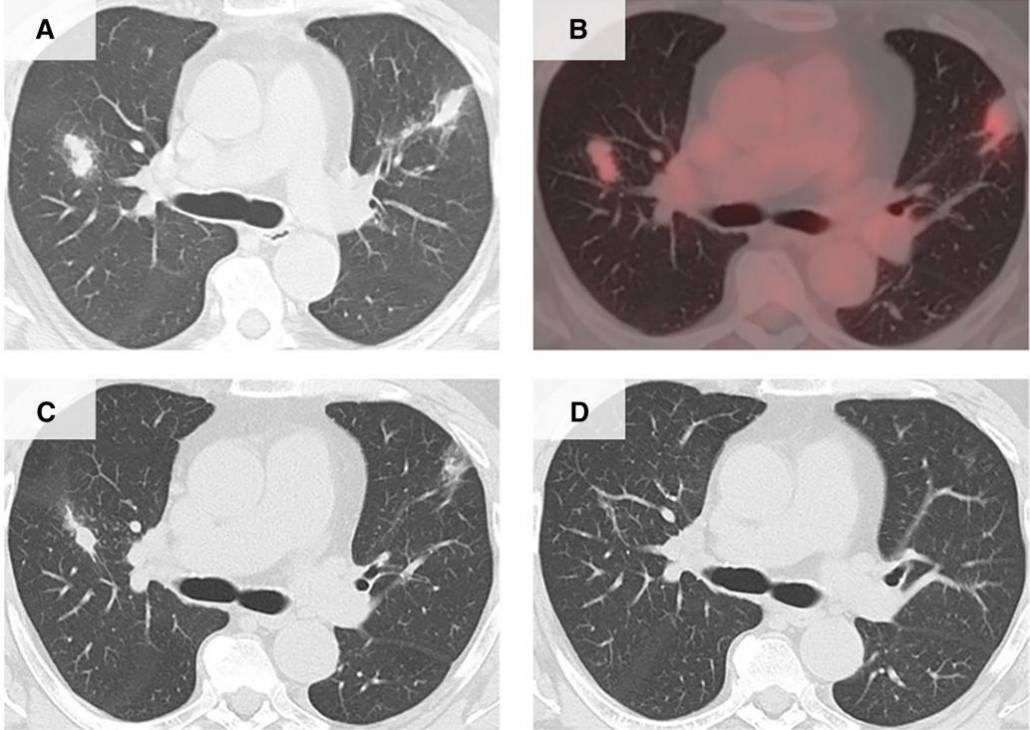
(**A**) Initial chest computed tomography (CT) showing ground-glass opacities in both lungs. (**B**) Positron emission tomography scan showing fluorodeoxyglucose uptake with a maximum standardized uptake value of 3.78 in the left lung lesion and 4.29 in the right lung lesion. (**C**) Follow-up CT after 2 months of corticosteroid therapy showing partial regression. (**D**) Follow-up CT 4 months after praziquantel treatment revealing near-complete resolution of the lesions.

Computed tomography-guided needle biopsy of a lung lesion revealed IgG4-positive plasma cell infiltration (200 cells/high-power field, IgG4+/IgG+ >40%) and lymphocyte and eosinophil infiltration without fibrosis or storiform changes, meeting the criteria for IgG4-related respiratory disease (IgG4-RD). Laboratory and imaging results showed minimal improvement after 2 months of prednisolone treatment (0.6 mg/kg per day) (Figure 1C). Based on history and findings, parasitic infection was suspected. Serology using multiple-dot ELISA revealed the presence of IgG antibodies against *Paragonimus westermani*, *Paragonimus miyazakii*, *Fasciola*, and *Clonorchis*. Inhibition ELISA revealed higher avidity for the *P. westermani* antigen than for the *P. miyazakii* antigen, confirming *P. westermani* infection. A retrospective histopathological review identified parasite eggs, confirming the diagnosis of pulmonary paragonimiasis (Figure 2).

**Figure 2.**
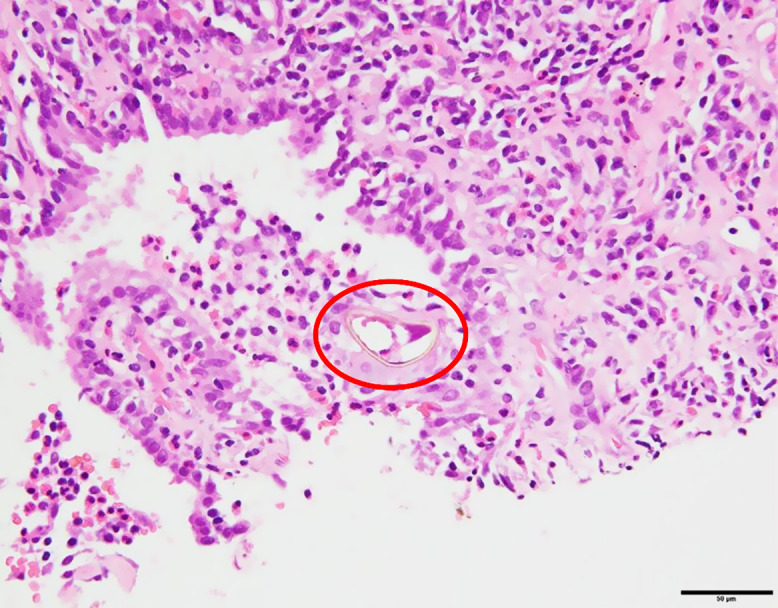
Hematoxylin and eosin staining of a lung nodule obtained by computed tomography-guided biopsy. The red circle highlights the parasite egg.

The eosinophilia resolved and IgG4 levels normalized rapidly after a 3-day course of praziquantel. The 4-month follow-up CT showed complete regression of the lung lesions (Figure 1D). Serological monitoring revealed a decline in both *P. westermani* and *P. miyazakii* IgG antibody levels, confirming successful treatment.

*P. westermani* is a parasitic trematode transmitted to humans through consumption of raw or undercooked freshwater crabs or wild game meat, primarily in Asia.^1^ In pulmonary paragonimiasis, chest CT typically shows bilateral peripheral nodules or infiltrative shadows, which need to be differentiated from those owing to lung cancer, tuberculosis, IgG4-RD, and eosinophilic pneumonia.^2^^–^^4^ Although *Paragonimus* eggs can be found in sputum, stool, or percutaneous lung biopsy specimens,^2^ direct identification is challenging, making serological testing crucial for accurate diagnosis.^1^

Pulmonary paragonimiasis shares clinical and histological features with IgG4-RD, often leading to misdiagnosis^3^ and inappropriate corticosteroid use, which can worsen the disease.^4^

This case highlights the need to avoid diagnosing IgG4-RD based solely on elevated IgG4 levels or increased IgG4-positive plasma cells without considering all of the histopathologic evidence. To prevent misdiagnosis, parasitic infections, such as paragonimiasis, should be considered in the differential diagnosis of suspected IgG4-RD.^5^

## References

[1] Kunitomo K Yumoto S Tsuji T, 2020. A case of paragonimiasis in a patient with wet cough. Am J Trop Med Hyg 103: 939–940. 32896236 10.4269/ajtmh.20-0395 PMC7470576

[2] Cong CV Anh TTT Ly TT Duc NM, 2022. Paragonimiasis diagnosed by CT-guided transthoracic lung biopsy: Literature review and case report. Radiol Case Rep 17: 1591–1597. 35309377 10.1016/j.radcr.2022.02.046 PMC8927937

[3] Saeki S Horio Y Hirosako S Ichiyasu H Fujii K Kohrogi H, 2015. Elevated serum IgG4 levels in two cases of paragonimiasis. Respirol Case Rep 3: 92–94. 26392854 10.1002/rcr2.110 PMC4571736

[4] Hashimoto T Nishizono A Hiramatsu K, 2025. A case report of pulmonary paragonimiasis diagnosed via the deterioration of pulmonary cavities during high-dose corticosteroid therapy for chronic eosinophilic pneumonia. Ann Thorac Med 20: 74–77. 39926405 10.4103/atm.atm_146_24 PMC11804950

[5] Wallace ZS Perugino C Matza M Deshpande V Sharma A Stone JH, 2019. Immunoglobulin G4-related disease. Clin Chest Med 40: 583–597. 31376893 10.1016/j.ccm.2019.05.005 PMC7133392

